# Linking flow-stream variability to grain size distribution of suspended sediment from a satellite-based analysis of the Tiber River plume (Tyrrhenian Sea)

**DOI:** 10.1038/s41598-019-56409-8

**Published:** 2019-12-19

**Authors:** J. Pitarch, F. Falcini, W. Nardin, V. E. Brando, A. Di Cicco, S. Marullo

**Affiliations:** 1 NIOZ – Royal Netherlands Institute for Sea Research, Department of Coastal Systems, and Utrecht University, PO Box 59, 1790 AB Den Burg, Texel The Netherlands; 2 CNR – Institute of Marine Sciences (ISMAR), Via Fosso del Cavaliere 100, 00133 Rome, Italy; 3 Horn Point Laboratory, University of Maryland Center for Environmental Science, Cambridge, MD 21613 USA; 4 ENEA – Centro Ricerche Frascati, Frascati, Italy

**Keywords:** Environmental sciences, Hydrology

## Abstract

Several coastal regions on Earth have been increasingly affected by intense, often catastrophic, flash floods that deliver significant amounts of sediment along shorelines. One of the critical questions related to the impact of these impulsive runoffs is “are flash floods more efficient in delivering non-cohesive sandy sediment along the coasts?” Here we relate flow stages (i.e., from erratic to persistent) to the grain size distribution of the suspended load, by performing a synergic analysis of *in-situ* river discharge and satellite-retrieved grain size distribution, from 2002 to 2014, covering the 2012 Tiber River (Italy) exceptional flood event. Our analysis shows novel and promising results regarding the capability of remote sensing in characterizing suspended sediment in terms of grain size distribution and reveals that erratic stages favour delivering of non-cohesive sandy sediment more than the persistent stages. This conclusion is supported by numerical simulations and is consistent with previous studies on suspended sediment rating curves.

## Introduction

**Figure 1:**
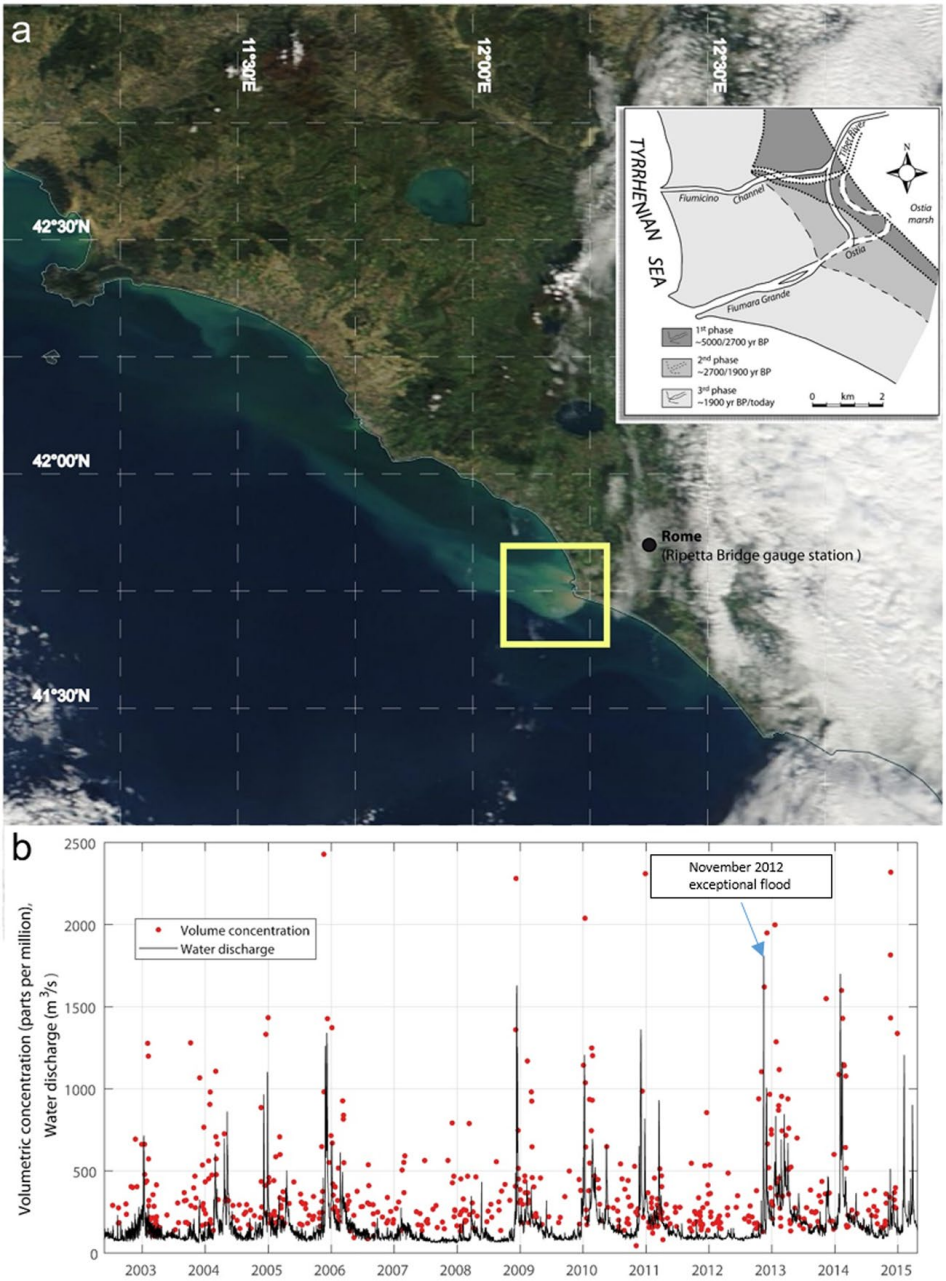
(**a**) MODIS Ocean Colour image on 17 November 2012 showing the suspended sediment plume; the yellow box around the Tiber River mouth represents the area where reflectances (Rrs) were extracted; the box shows the Tiber river delta evolution^2^. The image was created using data downloaded from the NASA (oceancolor.gsfc.nasa.gov) and processed using SeaDAS 7.2 (ref. ^55^); (**b**) Q_*w*_ and the total volume concentration of suspended particles (*VC*_tot_) time series (plot generated by using MATLAB 7.1 http://uk.mathworks.com/products/matlab); *VC*_tot_ is obtained from the sum of the two volume concentrations of the grain fractions considered in this work, i.e., 3.9 µm–62.5 µm and 62.5 µm – 125 µm.

The hydrologic regime of the Tiber River basin (central Italy; Fig. 1a) plays an important role in sediment transport and, in turn, in the evaluation of medium- and long-term changes of the Mediterranean coastline of central Italy, called Laurentine Shore by the Romans^1,2^. The Tiber River is the second largest river in Italy, with a length of 409 km and a drainage basin of about 17.156 km^2^; it is sourced from Central Apennines and debouches in the Tyrrhenian Sea, close to the city of Rome. Mean annual discharge of the Tiber River is about 225 m3/s; maximum annual discharge exceeds 1500 m^3^/s and the minimum annual discharge reaches 60 m^3^/s (ref. ^3^).

The Tiber River Delta is a progradating, wave dominated system, marked by a complex cusp made up of two distributary channels (Fig. 1). The last progradation cycle (still ongoing) has particularly intensified over the last 500 years and isolated the Roman Empire harbors from the sea^2^. Solid transport of the Tiber River is largely given by suspended load and only a small fraction by bed load^4^. In particular, the percentage of sediment load transported in suspension is more than 90% of the total amount of sediment load^3,5^. The average annual specific sediment load, measured from 1934 to 1973 at Ripetta Bridge, located in downtown Rome (43 km upstream the river mouth; Fig. 1), is about 290 ton/km^2^.

In November 2012, an exceptional flood brought the water discharge of the Tiber River to dangerous levels and it lasted from 13 to 15 November 2012 (ref. ^6^) (Fig. 1). The flood wave was originated from the exceptional river water flow due to the relevant precipitation in the upper part of the Tiber basin, which caused a significant water level rising between the November 14^th^ afternoon and 15^th^ morning in the lower part of the basin (Fig. 1). Several rain gauges recorded an abundant precipitation, higher than 300 mm/48 hours, in the upper and western Tiber watershed (Fig. 1). In the following days, a flood event propagated along the Tiber River main stream flow, reaching a peak in water discharge of 1,933 m^3^/s on November 15^th^ at 01:00 am (local time) at the water gauge station of Ripetta Bridge (Fig. 1). Floods like the one occurred in 2012 give us the opportunity to focus on amount and characteristics of the suspended load delivered from erratic events in respect to water discharge and its seasonality.

Inspired by the 2012 Tiber River flood, here we seek to quantify the impact of stream flow variability, i.e., erratic vs. persistent flow stages^7^, on river plume sediment assemblage, by the analysis of grain size distribution (GSD) of the suspended sediment, retrieved from satellite. Grain size plays a fundamental role in settling velocity and dispersal of suspended sediment over shelf areas^8,9^. Observations showed that formation of sedimentary bottom layers and their related sandy and/or mud deposits strongly depends on sediment (sand, silty and/or clayey particles) settling properties^10–13^. In particular, the role of sediment cohesion, which is controlled by sediment size, influences morphology of deltas and coastal areas^14^.

Although the knowledge of sediment characteristics gives a substantial contribution in understanding and modeling particle dynamics and coastal geomorphology, GSD of suspended particles from river plumes are still seldom quantified and insufficiently documented. Satellite data can be used as an alternative technique^15^, in particular, off river mouth and estuarine environments, where remote sensing algorithms can be applied to mid-resolution ocean color sensors (e.g., MERIS, MODIS, SeaWiFS, VIIRS, and OLCI) that provide near-daily data at ~1 km spatial resolution since two decades. Moreover, remote sensing has the additional advantages of providing a synoptic view of river plumes^16–20^. Application of *in-situ* calibrated MODIS satellite data, for instance, allowed capturing the spatio-temporal variability of the distribution of suspended sediment over the coral reefs^21^ and assessed the impact of these extreme outflows on wetland sedimentation during the historic 2011 Mississippi River flood^18^.

The relationship between the spectral beam attenuation coefficient (c_p_) and the GSD has been predicted theoretically and verified experimentally by many studies^22–24^. However, those findings had no application to remote sensing data since the beam attenuation coefficient is not retrievable from space. Fortunately, after commercial optical backscattering meters became available, research provided evidence that the spectral particle backscattering coefficient (b_bp_) and the GSD are also related^15,25–28^ in a very similar fashion as c_p_ and the GSD were found to be related. The advantages of this finding are many because b_bp_ can be retrieved from ocean color observations, thus establishing a link between remote sensing measurements and the spatio-temporal variability of particle assemblage (i.e., the more or less large grain size fraction) for fluvial geomorphology applications and, in particular, for understanding and monitoring fate and distribution of riverine suspended load characteristics. All this sets the base for our investigation on flow-stream variability and GSD of suspended sediment from a satellite-based analysis off the Tiber River mouth.

## Data and Methods

### *In situ* hydrological data

Along the Tiber River, the water level is measured at several river flow gauges combining water level and empirical river discharge. In this work we use Tiber River daily water discharge (*Q*_*w*_) data at the historic Ripetta flow gauge, matching the period of satellite observations. Ripetta’s station (http://www.idrografico.roma.it), has one of the most complete measurements data set monitoring the river discharge relative at more than 96% of the Tiber River drainage basin. To estimate the streamflow variability we then calculate monthly averages from the daily dataset, as well as the coefficient of variation of streamflow, $CV=\frac{{\sigma }_{Qw}}{\overline{{Q}_{w}}}$, where $\overline{{Q}_{w}}$ is the flow discharge monthly mean and ${\sigma }_{Qw}$ its standard deviation^18^; low (high) *CV* indicates flow regimes that are weakly (highly) variable around the mean and are termed as persistent (erratic). The use of monthly averages allows us to highlight the link between GSD of suspended sediment off the river mouth and the streamflow variability^7^. From the comparison between daily water discharge and *CV* (see Fig. S1 in the Supplementary Information) we observe that the coefficient of variation marks, properly, those months that were characterized by strong variability (i.e., erratic flows).

### Remote estimation of the backscattering coefficient

Light back-scattered by marine particles depends on size distribution, concentration, refractive index, and the detection wavelength^28–30^. For a poly-dispersed assembly of non-absorbing, spherical particles with a Junge-like size distribution (see Eq. 4 below), this dependency is represented by the power law approximation of the spectral particle backscattering coefficient

1 $${b}_{bp}(\lambda )={b}_{bp}({\lambda }_{0}){(\frac{\lambda }{{\lambda }_{0}})}^{-\eta }\,$$

where *λ*_0_ is a reference wavelength and *η* the spectral slope (dimensionless).

Here we retrieve the b_bp_ from remote-sensing reflectances (R_rs_) data, by using the quasi-analytical algorithm (QAA) (ref. ^31^), an approach that was validated in a previous work for Italian coastal and open waters^32^. In particular, we use observations from the Moderate Resolution Imaging Spectro-radiometer (MODIS) for the period 2002 to 2014, i.e., a data record that includes the November 2012 flood event (see Text S1 in the Supplementary Information). R_rs_ data were remapped to a ~ 20 x 20 km2 box centered around the Tiber River mouth (Fig. 1). Its size was chosen to be 2–3 times greater than the river-induced Rossby radius of deformation in order to ensure that the whole river plume bulge was considered in our analysis^33,34^.

The QAA parameterization was left as originally documented except for *η*, a parameter that is highly relevant for the GSD estimations. In particular, the default value is given by^35^:

2 $$\eta =2\{1-1.2\,\exp \,[-0.9\frac{{r}_{rs}(443)}{{r}_{rs}(555)}]\}$$

where $${r}_{rs}={R}_{rs}/(0.52+1.7{R}_{rs})$$.

**Figure 2:**
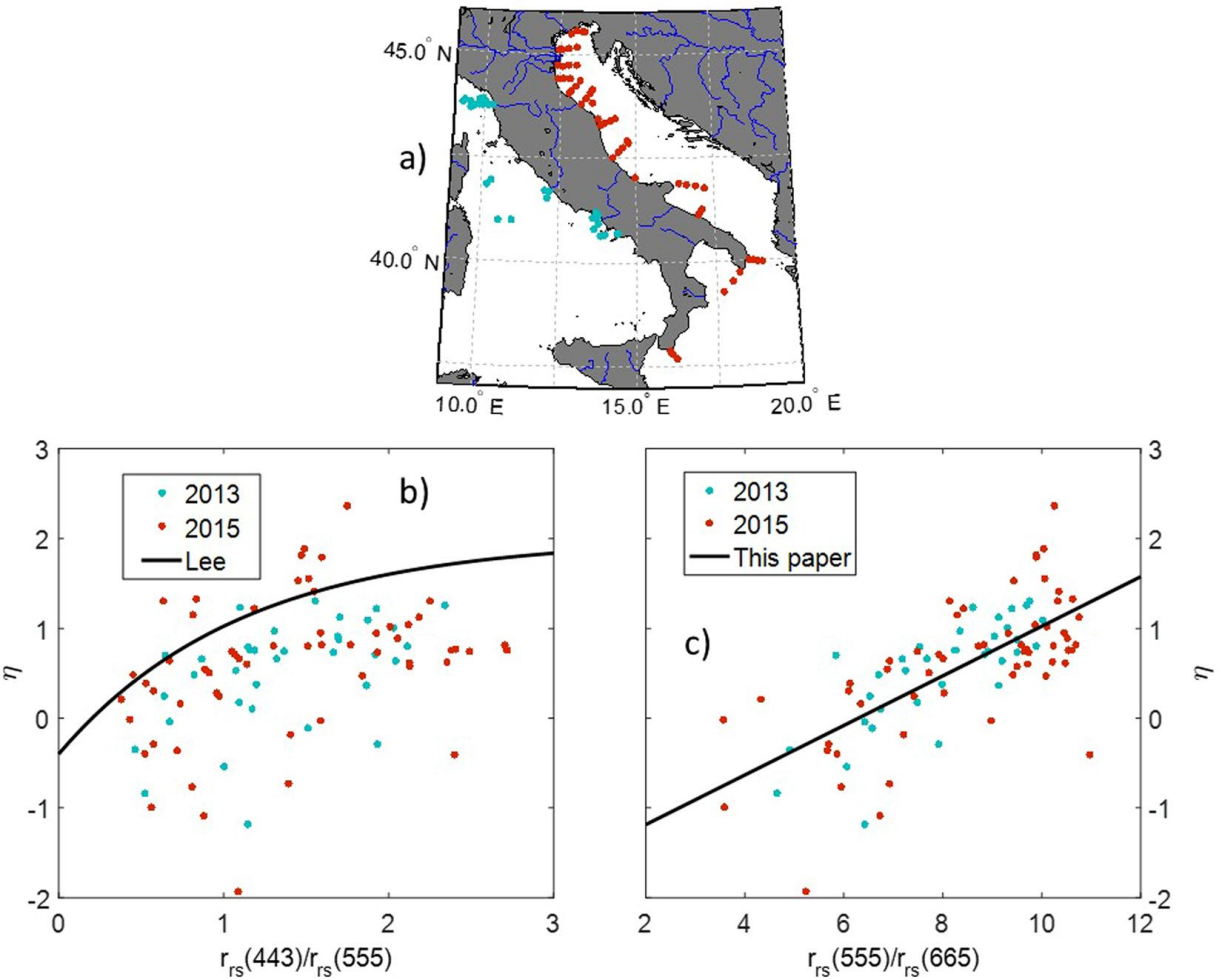
(**a**) Geographical distribution of the *in situ* R_rs_ and b_bp_ data. (**b**) r_rs_(443)/r_rs_(555) vs. *η* and Lee’s curve (ref Lee), RMSE = 0.93 m^−1^. (**c**) r_rs_(555)/r_rs_(665) vs. *η* and the best linear fit for the data: η = 0.2764 r_rs_(555)/r_rs_(665)−1.793, r^2^ = 0.48, RMSE = 0.51 m^−1^. Plots are generated by using MATLAB 7.1 http://uk.mathworks.com/products/matlab.

Equation (2) was originally built by using *in-situ η* vs. r_rs_(443)/r_rs_(555) matchups (see below) but the data cloud was highly scattered around the mean regression curve^31^ (Fig. 2b), thus having little predictive value; additionally, data were mainly taken from open ocean waters. A biased estimated *η* does not have a huge impact on the retrieval of absorption and its various components, which is for what the QAA is mostly used for, but here *η* is crucial component of the retrieval.

Hence, to provide reliable *η* estimates, we analyzed matched *in-situ* spectra of (R_rs_) and b_bp_ at various stations, covering a wide turbidity range, belonging to two field campaigns, conducted in 2013 and 2015 across Italian seas (Fig. 2a). b_bp_ was measured with an ECO-VSF3 (Wet Labs, Inc.) at the wavelengths 470, 530 and 660 nm. From surface data, the spectral slope (*η*) was calculated as the slope of the best linear fit of b_bp_ against the wavelength, both log-transformed. R_rs_ was measured using the free-profiling technique using OCR-507 radiometers (Satlantic, Inc.), at the bands 412, 443, 490, 510, 555, 665 and 863 nm using protocols compliant with NASA’s recommendations^32,36,37^.

Figure 2b shows the blue-to-green band ratio r_rs_(443)/r_rs_(555) from our *in-situ* reflectances respect to our measured *η*. Data shows high scattering but also high overestimation respect to Lee’s curve (Eq. 2). For this reason, we choose the ratio r_rs_(555)/r_rs_(665) as predictive variable, which results to be more sensitive to variations in coastal waters; indeed, the fit is largely improved (Fig. 2c). The best linear fit to this latter data cloud is the equation that we propose here to estimate the backscattering spectral slope from remote sensing, where we have replaced the *in-situ* band 665 nm with the MODIS band 667 nm, assuming negligible difference between them:

3 $$\eta =0.2764\frac{{r}_{rs}(555)}{{r}_{rs}(667)}-1.7393.$$

### GSD power law approximation

Generally, simple power law approximations of GSD (Eq. 3) can be successfully used to describe and assess poly-dispersed particle assemblies in a wide array of oceanic and estuarine water types and river plumes^28,38^.

4 $$N(D)=N({D}_{0}){(\frac{D}{{D}_{0}})}^{-\xi }$$

where *D*_0_ is a reference particle diameter and *ξ* is a dimensionless parameter called Junge slope^39^. A higher *ξ* implies a higher proportion of smaller particles and vice-versa^40^. *ξ* values generally increase from river water to the open ocean^38^, clearly indicating that coarser particles are more confined to the near-shore areas.

The full size range can be divided in several sub-ranges to study the proportion between size classes. For instance, Kostadinov *et al*. (ref. ^25^) applied this approach to study the particle abundance and volume in the dimensional ranges corresponding to three main phytoplankton-size classes in the global ocean^40,41^. Here, we consider the silt range, between the limits 3.9 µm and 62.5 µm, and the very-fine sand range, between 62.5 µm and 125 µm.

By assuming spherical shape, the volume concentration (*VC*) between two given edge sizes (*D*_min_*, D*_max_) is equal to:

5 $$VC={\int }_{{D}_{min}}^{{D}_{max}}N(D)\frac{4}{3}\pi {(\frac{D}{2})}^{3}\,dD.$$

*VC* estimation can relate to a size fraction like silt (3.9 µm to 62.5 µm), sand (62.5 µm to 125 µm) or to the global assembly by taking the full size range (3.9 to 125 µm). In this latter case, it is named total volume concentration (*VC*_tot_). For these two size classes *VC*_tot_ is equal to the sum of the particle volume of each class, reported in parts per million^28^.

A useful parameter, related to a GSD, is the median diameter (D50), which is the diameter at which the lower and the upper volume fractions of the GSD are equal. It is obtained after the integration of the GSD in Eq. (5), following the Junge assumption in Eq. (3):

6 $${D}_{50}=\{\begin{array}{c}{(\frac{{D}_{max}^{4-\xi }+{D}_{min}^{4-\xi }}{2})}^{\frac{1}{4-\xi }}\,,\,\xi \ne 4\\ \sqrt{{D}_{min}{D}_{max}}\,,\,\xi =4\end{array}\,$$

### Remote estimation of the GSD from the backscattering coefficient

Theoretical derivations that used the Mie scattering theory^25^ and experimental investigation^26,27^ showed that the GSD can be associated to b_bp_ by means of *η* in Eq. (1). Such research built on previous knowledge that focused on the beam attenuation coefficient c_p_ instead of on b_bp_ (refs. ^23,24,29^). Kostadinov *et al*. (ref. ^25^) found that if b_bp_ was predicted using Mie calculations, a relationship between *η* and *ξ* also existed, and was similar to that related to c_p_. Theoretical and empirical studies agree on the fact that *ξ* increases as a monotonic function of *η* and thus, as for *ξ*, lower *η* is associated to a higher proportion of large particles and vice-versa.

In this work, we use the look-up table (LUT) provided by Kostadinov *et al*. (ref. ^25^). The LUT requires the input of the spectral b_bp_, which is estimated with the QAA. The LUT returns the Junge slope *ξ*, and also the GSD value N_0_ at a reference size D_0_, which is relevant for *VC*_tot_ calculations. In this regard, we notice that *VC*_tot_ is a concentration value of particles within a given size range, but is conceptually and methodologically different than the usual suspended particulate matter concentration which is commonly retrieved by using remote sensing techniques and expressed in mass density units (e.g.^42–44^).

### Remote and hydrological data alignment

To track the river’s turbid water off the river mouth from satellite-based images, we select the maximum R_rs_(667) value within the box for each day (Fig. 1a). In the practical implementation, we chose the sampled pixel as the closest to the 99.5th percentile instead of the maximum to avoid outliers due to inaccuracies in pixel flagging, usually along the coastline or close to clouds. We thus obtained a time series of daily R_rs_ spectra (at 443, 488, 555 and 667 nm) over the same period of the collected water discharge data. From this daily time series, daily time series of *η* and the GSD were obtained and then monthly averaged.

### Numerical experiments

**Figure 3:**
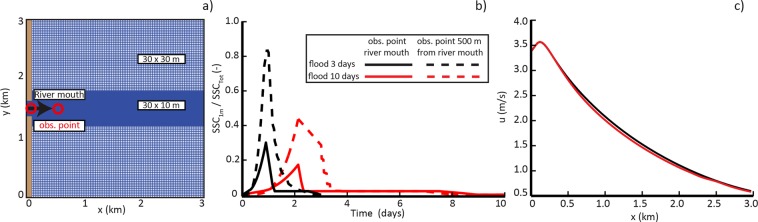
(**a**) Cells size distribution of the computational domain. Red circles show observation points position at the river mouth and 500 m from the river mouth. (**b**) Suspended sediment concentration ratio increases and then decreases during flood events. Colored lines display different duration of floods while continuous line and dashed shows, respectively, the propagation sediment at the river mouth and at the distance of 500 m. The location of the observation points is highlighted by red circles in the panel (a). (**c**) Along-stream velocity profile for the two flood discharges we consider in our work: 3-day flood (blue solid line) and 10-day flood (red dashed line). The downstream transect begins from the channel mouth and is positioned at the half channel width. Plots are generated by using MATLAB 7.1 http://uk.mathworks.com/products/matlab.

We model river mouth sediment dynamics with Delft 3D^45,46^, by using different flood discharge evolution. In particular, we test consistencies between a model of sediment transport based on remote sensing observations, and a generic numerical model of sediment transport, by exploring the relation between GSD and stages (erratic vs. persistent) of river outflows (Fig. 3). Delft 3D simulates fluid flow, waves, sediment transport, and morphological changes at different timescales. The equations of fluid, sediment transport and deposition are discretized on a 3D curvilinear finite difference grid and solved by an alternating direction implicit scheme. Here we use the three-dimensional formulation of the hydrodynamic and sediment transport models, implemented in Delft 3D.

Delft 3D solves the Navier-Stokes equations for an incompressible fluid with the assumptions of shallow water, Boussinesq, and hydrostatic approximation. The standard *k-ε* closure model^47^ is used for the vertical eddy viscosity, and the horizontal eddy viscosity is computed with a large eddy simulation technique. Transport of suspended sediment is calculated by solving the three-dimensional Advection-Diffusion equation, computed by following the method of Van Rijn^48^.

For our simulation, the numerical domain is a square (3 km × 3 km), in which there is a river flow in inlet on the western side (Fig. 3a). The computational grid is composed of squared cells (30 m × 30 m) but, along the centerline, a refinement occurs, where each cell size is 30 m × 10 m. We simulate a different flood progression with linear growth and falling limb two times, compared to the rising limb but same peak discharge, i.e., *Q*_*w*_  = 2000 m^3^/s. For both study cases, we set up a rising discharge *Q* and suspended sediment concentration (*SSC*) entering in the domain from the river mouth (Fig. 3a). We remark that even though the two flood discharge evolutions we consider in the numerical experiment are different, they are characterized by similar along-stream velocity profiles (Fig. 3c). In particular, the two velocity profiles remain identical till the first 500 m downstream, from the channel mouth (where the GSD analysis is performed). Moreover, we make clearer that our choice is not “coincidental”: the 3-day flood and a 10-day flood were designed to demonstrate that the ‘erraticity’ (rather than the velocity) is responsible for the different suspended sediment GSD we observe from remote sensing.

For the simulations we consider the upper limit of very-fine sand size range (i.e., 125 μm), according to the fact that the suspended fraction recorded during the Tiber River floods ranges from clay to very fine sand (3.9–125 µm) and that the non-cohesive very fine sand fraction is the most predominant one^3,4^. At the inlet, we impose a constant input of 0.6 kg/m^3^ of suspended sediment concentration^49^. All sediment is characterized by specific density of 2,650 kg/m^3^ and dry bed density of 1,600 kg/m^3^. On the north, south, and eastern boundary we assigned a water elevation equal to zero (Fig. 3a) to reproduce the sea level. A trapezoidal river channel with flow depth *h* = 3 m is incised into a non-erodible coastline, where we imposed a zero water depth and no flux boundary condition. The bottom roughness is modeled with the Chézy’s formulation, using the constant Chézy coefficient value (i.e., 65 m^1/2^/s). The initial condition of the models consists of a constant bathymetry with 5 m of erodible sediment on the basin bottom and a time step *Δt* = 9 s.

## Results

Correlation between water discharge (*Q*_*w*_) and the satellite-based total volume concentration of suspended particles (*VC*_tot_) shows a comforting agreement, i.e., *r* = 0.55 (Fig. 1b); peaks of water discharge are always associated with high values of *VC*_tot_, which ranges from 1200 to 2500 ppm. This qualitatively suggests that the remote sensing approach, along with the reflectance sampling method we implemented, is a promising tool for complementing the *in situ* hydrological dataset.

**Figure 4:**
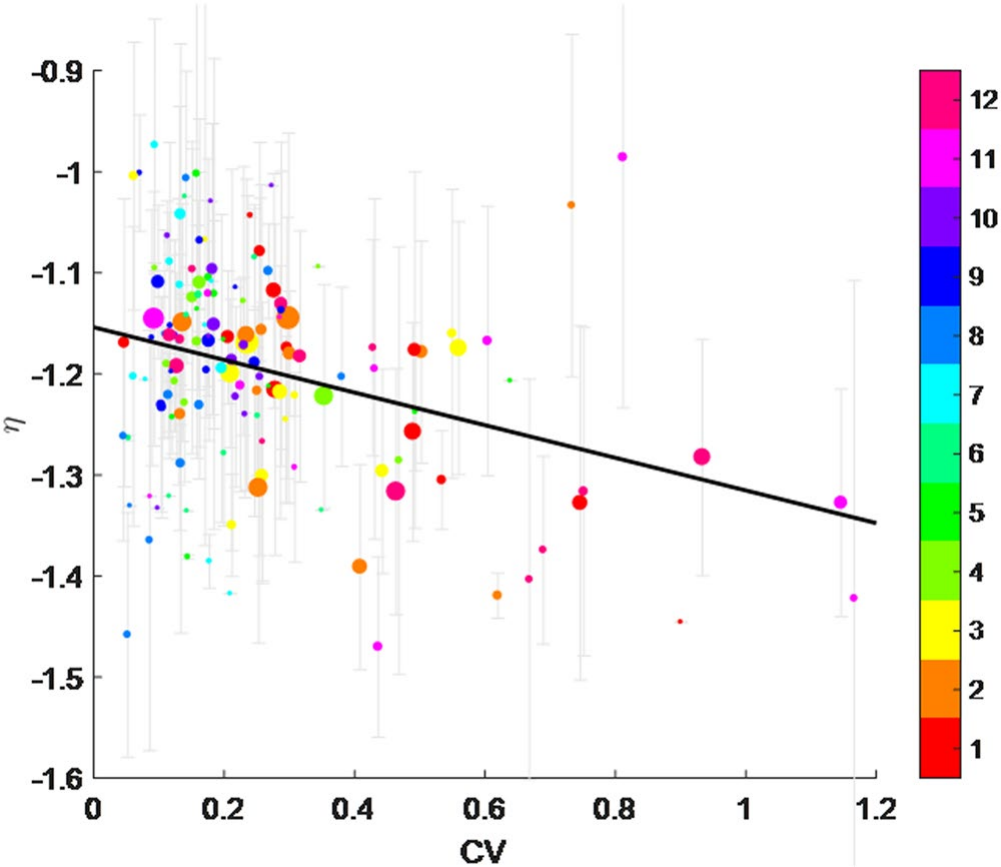
Scatter plot of the coefficient of variation of monthly river discharge (CV) vs. the monthly particle backscattering spectral slope (*η*). Both variables are dimensionless (see text). The standard deviation of monthly *η* is also indicated as vertical bars; dot diameter is proportional to the number of cloud-free satellite images. The best linear fit (*y* = −0.186 *x* -1.143; r^2^ = 0.35) is meant to highlight the general trend that marks the relation between flow erraticity (CV) and GSD. Months with less than three valid observations were discarded, as calculation of a standard deviation is not possible for them. Colorbar indicates months for each point (month numbers are also reported); reddish dots represents late fall/early winter months. Plot generated by using MATLAB 7.1 http://uk.mathworks.com/products/matlab.

The synergy between remote sensing and hydrologic measurements, specifically, reveals an intriguing relationship between flow stage characteristics (i.e., flow erraticity) and the mean grain-size population. Here, we use the monthly particle backscattering spectral slope (*η*) as an indicator of the GSD. By plotting the monthly *η* versus the coefficient of variation (CV) we find that CV and *η* correlate negatively, thus suggesting that more erratic river discharges (higher CV) relate to a larger proportion of large particles in suspension. On the contrary, more persistent flow discharges (i.e., low CV) are related to finer particles in suspension (Fig. 4). Although the dot cloud of this correlation is rather disperse, this general trend is highlighted by the best linear fit: for a given water discharge, grain-size of the suspended sediment will be larger if the flow is more erratic than if the flow is persistent. Moreover, focusing on seasonality, the correlation shows that erratic events occur mostly in late fall/early winter, when we observe high proportion of large particles in suspension.

**Figure 5:**
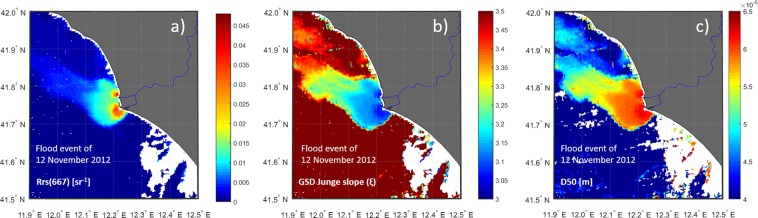
(**a**) Rrs(667) [sr^−1^] as a proxy for suspended sediment; (**b**) GSD Junge slope; (in situ) median particle diameter (D50) [m] of the GSD, after assuming a Junge slope with a minimum size of 3.9 µm and a maximum of 125 µm, for the 12 November 2012. The D50 [m] is analytically obtained by assuming a Junge-type GSD between the range 3.9–125 µm and it is zero out of this range. The three panels confirm that the proportion of smaller particles increases from river mouth to the open ocean and thus that coarser particles are more confined to the near-shore areas. Plots are generated by using MATLAB 7.1 http://uk.mathworks.com/products/matlab.

To analyze consistency of our results, we evaluate the Junge slope (*ξ*) and the median of grain-size diameters (i.e., the D50) within the plume, for the day that records the maximum concentration of suspended sediment of the whole time series off the river mouth, i.e., the flood event of 17 November 2012 (Fig. 5). It results that R_rs_(667), which is a good proxy for turbidity, sharply decreases with the distance from the river mouths (Fig. 5a); Fig. 5b,c refer to particle quality more than quantity, which explains why the plume extent is better delineated than in panel a). Still, particle sedimentation affects GSD within the river plume. Largest, presumably non-cohesive particles (i.e., D50 ~ 65 µm) remain in suspension for few hundreds of meters from the two main mouths of the Tiber River, while the finest (clay and silt) fraction is delivered off shore. Therefore, within the plume, Fig. 5b,c shows how the grain size is decreasing as the distance to the coast increases.

The numerical experiment confirms the link between erraticity of the outflow and its ability to suspended higher concentration of sediment. To assess the behavior of suspended sediment in the river jet plume for different flow stage conditions, we obtain suspended sediment concentration for the entire water column, *SSC*_TOT_, and at 1 m below the water surface, *SSC*_1m_, from numerical simulations. The ratio *SSC*_1m_ / *SSC*_TOT_ is assumed to represent the ability of the outflow to keep large (i.e., 125 μm) grain-size in suspension, and thus, to be comparable with the satellite-retrieved Junge slope. Numerical results show that, although reaching the same value of water discharge, and despite the fact that the two run show similar along-stream velocity profiles, the erratic event (i.e., the 3-days flood in Fig. 3b) suspends larger concentration of very fine-to-fine sediment, compared to a more persistent event (i.e., the 10-days flood). Moreover, in both study-cases the sediment concentration in the upper part of the plume increases due to the jet expansion. In fact, most of the sediment is suspended from the bottom layers, as bedload, to the top.

## Discussions and Conclusions

Analysis of matched remote-sensing and *in-situ* hydrological data, supported by numerical simulations, showed that streamflow variability (CV) correlates with the grain size of the suspended sediment. Accordingly, Basso *et al*. (ref. ^7^) found that large values of coefficient of variation (CV), are associated to large values of the *δ* -coefficient of the sediment rating curve, i.e., the power law relation between water and sediment discharges, ${Q}_{s}=\beta {Q}_{w}^{\delta }$ (where 𝛿 and *β* are empirical coefficients)^50^. In turn, the *δ*-coefficient represents the erosive power of the river, with large values being indicative for rivers with a strong increase in erosive power and in sediment transport capacity when discharge increases^38^. Moreover, *δ*-values are also affected by the GSD of the material available for transport, i.e. in streams characterized by sand sized sediments the power of the stream to transport sediment will be more important than in streams that mainly transport silt and clay^51^ (Walling *et al*.^40^); this will result in high *δ*-values^52^.

Our results show that the synergy of remote sensing and hydrologic *in situ* data expands and complements these previous findings from two aspects: i) the satellite-based reconstruction of GSD (or particle backscattering spectral slope) time series allows for long-term statistical analyses of both water discharge and suspended sediment load, thus complementing and integrating GSD *in situ* measurements; ii) the synoptic property of satellite images allows for a spatial view of the sedimentary characteristics of the plume, thus helping the recognition of river plume pattern in terms of GSD. Indeed, by analyzing a ten-year-long time series, we assess the ability of unsteady flow to transport large particle size in suspension in a real field case^53^. Moreover, by focusing on the 2012 erratic event, we pictured depositional patterns of different grain size off the river mouth, a crucial feature that may largely impact shoreline restoration strategies^54^.

Previous researches demonstrated the possibility of using satellite to retrieve GSD^24–27^. Based on those, we retrieved a GSD long time series within the Tiber River plume off the river mouth. The resulting analysis, which paired river flow discharge data to satellite-based GSD showed i) an encouraging correlation (r = 0.55) between water discharge (*Q*_w_) and the total volume concentration (*VC*_tot_) of suspended particles and ii) a relationship between flow stage characteristics (i.e., flow erraticity) and the spectral slope of backscattering (η), in turn, related to the GSD. Finally, by running a numerical model that confirmed what we obtained from remote sensing, therefore, assessing the relationship we find between GSD and flow variability. This, in turn, gives also robustness to our ansatz, that is, the methodology to retrieve GSD from satellite spectral slope of backscattering.

Unsteady flow makes the problem of sediment transport very complex and difficult to describe in general laws. Our work presents a novel, satellite-based tool for retrieving GSD of suspended solids debouching from river mouth, by adapting a semi-analytical technique^25,26^ to coastal geomorphology and sediment transport applications. From the new satellite-based tool, we find an emerging relation between GSD and flow stages (erratic vs. persistent floods), also relating this to seasonality. By setting an ad hoc numerical experiment, we then provide a mechanistic support to our findings, setting novel hypotheses on the role of sharp spatial pressure gradient (rather than shear velocity) in remobilizing sediment during river floods. This demonstrates the geomorphological effectiveness of flash floods, which are able to deliver fine-to-medium suspended load off river mouths.

## Supplementary information

Supplementary Information
