# Supplementary material for: Linking flow-stream variability to grain size distribution of suspended sediment from a satellite-based analysis of the Tiber River plume (Tyrrhenian Sea)
Source: Sci Rep. 2019 Dec 19;9:19729. doi: 10.1038/s41598-019-56409-8 (PMC6923369; doi:10.1038/s41598-019-56409-8)
Supplement: Supplementary file 1 — Supplementary Information [file 41598_2019_56409_MOESM1_ESM.docx]

**Supplementary Information for** **Linking flow-stream variability to grain size distribution of suspended sediment from a satellite-based analysis of the Tiber River plume (Tyrrhenian Sea)**

J. Pitarch^1^, F. Falcini^2*^, W. Nardin^3^, V. E. Brando^2^, A. Di Cicco^2^, S. Marullo^2,4^.

*^1^ NIOZ – Royal Netherlands Institute for Sea Research, Department of Coastal Systems, and Utrecht University, PO Box 59, 1790 AB Den Burg (Texel)*

*^2^ CNR – Institute of Marine Sciences (ISMAR), Via Fosso del Cavaliere 100, 00133 Rome, Italy*

*^3^ Horn Point Laboratory, University of Maryland Center for Environmental Science, Cambridge, MD 21613, USA*

*^4^ ENEA – Centro Ricerche Frascati – Frascati, Italy*

**Introduction**

This Supplementary Information provides additional details on:

• Comparison between daily water discharge and the coefficient of variation (Fig. S1);

• Estimation of the satellite remote sensing reflectance (Text S1).


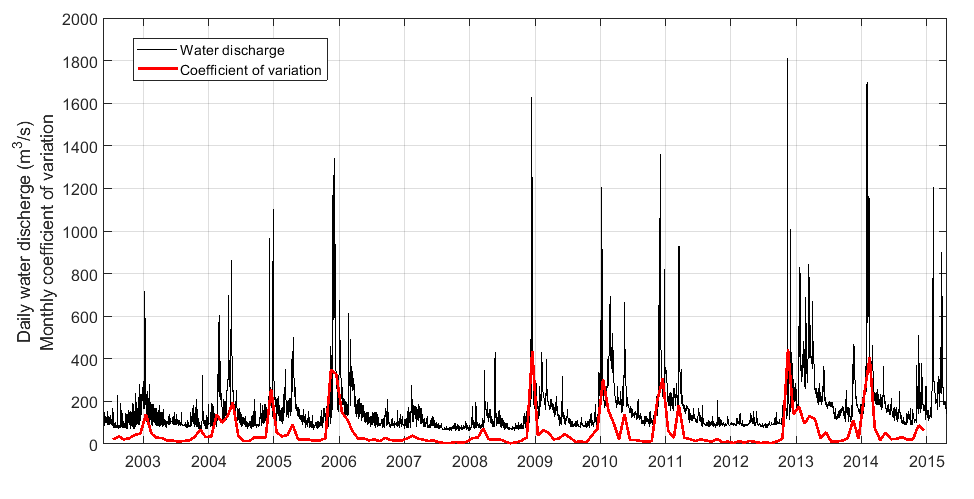


**Fig. S1**. Comparison between daily water discharge and the coefficient of variation

**Test S1: Estimation of the satellite remote sensing reflectance**

Moderate Resolution Imaging Spectro-radiometer (MODIS) AQUA L1A images were downloaded from the National Aeronautics and Space Administration (NASA) “Ocean Color data” web page (https://oceandata.sci.gsfc.nasa.gov/) over the area for the period 2002 to 2014. L1 files are required to apply atmospheric corrections more suitable in retrieving water reflectance in river plume waters, where the standard near-infrared region (NIR) algorithm tends to fail^1,2^. Then, L2 data, made of atmospherically-corrected remote-sensing reflectances (R_rs_), were produced with the Seadas 7.0 software, by means of l2gen command. Processing options were the default ones, except for the aerosol mode, which was set to “multi-scattering with 2-band model selection and short wave infrared (SWIR) correction” (ref.^3^). This algorithm, preferred for highly turbid waters, switches between the bands 1240 and 2130 nm as a function of the Water Turbidity Index, which represents a first estimation of water turbidity based on the 748 and 1240 nm spectral bands. Furthermore, it was found that keeping the cloud threshold to the default value of 0.027 reduced data availability even for cloud free or glint free conditions^4^, so it was lowered to 0.018, following an approach similar to the one pursued by Dogliotti et al. (ref. ^2^). This set-up method provided reliable output R_rs_ even over very turbid waters next to the river mouths.

**References**

Wang, M., Son, S., & Shi, W. (2009). Evaluation of MODIS SWIR and NIR-SWIR atmospheric correction algorithms using SeaBASS data. Remote Sensing of Environment, 113(3), 635-644.

Dogliotti, A. I., Ruddick, K., Nechad, B., & Lasta, C. (2011). Improving water reflectance retrieval from MODIS imagery in the highly turbid waters of La Plata River. In Proceedings of the VI International Conference Current Problems in Optics of Natural Waters (ONW’2011), St. Petersburg, Russia (pp. 6-9).

Wang, M., and W. Shi (2007), The NIR-SWIR combined atmospheric correction approach for MODIS ocean color data processing. *Optics Express*, 15(24), 15722-15733.

1. Liang, Q., Zhang, Y., Ma, R., Loiselle, S., Li, J., & Hu, M. (2017). A MODIS-based novel method to distinguish surface cyanobacterial scums and aquatic macrophytes in Lake Taihu. *Remote Sensing*, *9*(2), 133.
